# Some Observations on Chorea

**Published:** 1829-10-01

**Authors:** 


### XXIII.

### Some Observations on Chorea.

That chorea is only one of the thousand forms which hysteria assumes we have no doubt. But we suspect that one very important, and not very uncommon, feature of the complaint generally escapes the notice of the medical practitioner—or rather it is studiously concealed from his view. He sees plainly enough, the morbid movements of the muscles; but he knows not the train of disordered, sometimes frightful, ideas that are passing in the mind. In three successive cases of severe and long-continued chorea which lately came under our notice, the irregular and distressing muscular movements were but as a feather in the scale of suffering, when compared with the terrific images that sickened or overwhelmed the soul. These are often, or rather always, most carefully concealed. It was by mere accident that we discovered them in the first of the three cases; but by dint of persevering inquiry we wormed them out of the other two, though with the greatest difficulty. In all three, the mental impressions are pretty uniformly in proportion to the corporeal disorders. In all three, there were moral causes in early operation, and evidences of bodily derangement. But whether the moral produced the corporeal disorder, or the latter the former, we cannot discover. Certain it is, that the mental impressions always exasperate the choreal or hysterial phenomena—and that any increase of the bodily ailments (we mean of the chylopoietic derangements) invariably brings on the terrors of the mind. What these last are, we do not hold ourselves at liberty to say. They are various in kind and also in degree. Some of them are ludicrous—some ridiculous—but most of them are horrible or unnatural. In none of these three cases did the patients ever broach the subject of their mental maladies to even their nearest relations—nor, indeed, to their medical attendant, but they were drawn from them by repeated inquiry. This leads us to suspect that the mental phenomena much more frequently accompany chorea and hysteria than is commonly imagined.

The difficulty of curing the disease may be, in part, accounted for by this concealment of more than half the complaint—and by the misery of concealment itself. And even when we become acquainted with the secret grievance, we can only act through the medium of the body.

An interesting case of this disease has lately been stated in the Glasgow Journal by Dr. Harrower, which is worthy of notice. The patient was a slender and delicate girl, ten years of age, who had been three months affected with chorea before he saw her. The left side was principally affected.

"She cannot with the left hand take hold of any object without inducing a train of irregular involuntary motions. Even when the object is grasped for a moment, she is unable to retain it. The flexor and extensor muscles seem to act alternately, giving rise to many whimsical gestures. Appetite craving; tongue clean; bowels sluggish; evacuations not formed, and of a light brown colour; pulse frequent, weak and intermitting; body considerably emaciated. She tosses about in bed continually, frequently sitting up and turning her body as on a pivot in a semirotatory manner. At times, the motion is confined to the head, which turns so quickly, that she actually seems to be looking in all directions at the same moment. Occasionally the extensor and flexor muscles of the body come into action. She continues sitting, throwing the body backwards on the bed and forwards on her knees, after which the paroxysms generally terminate by great exhaustion of strength. There is weeping, alternating with fits of involuntary laughter. Her hands are continually in motion, sometimes apparently endeavouring to lay hold of objects floating before her eyes, at other times pinching the bedclothes and even her own body, or her attendants. Her speech is very indistinct, with a tremulous motion of the tongue. She has slept none for the last eight days, except a few minutes at the termination of the paroxysms, when she appears to drop into a slumber from exhaustion, but is generally roused by the recurrence of the fits. These are evidently aggravated by attempts to restrain her. Countenance vacant; pupils very much dilated and sluggish in their motions; vision dim; dulness of hearing; intellect much impaired. She has been unable to feed herself for some weeks, and has much difficulty in swallowing, the morsel being frequently ejected with considerable force from the mouth by the spasmodic contraction of the muscles of the pharynx. On examination, the spine was found tender along its whole course, particularly about the ninth and tenth dorsal vertebræ. When these were pressed pain was felt in the left hypochondriac region which was somewhat distended; and her mother had observed that she could not allow her clothes to be drawn so tight as usual. She had used no remedies except some laxatives."

A course of medicine, with the cold bath and dry friction, was prescribed, which was continued a fortnight without benefit. The evacuations were depraved; the involuntary motions continued to increase; there was pain of the occiput, difficulty of deglutition, loss of speech, ghastly countenance, fixed eyes, foaming of the mouth and grinding of the teeth. She became furious in the extreme, climbing with the agility of a monkey and getting many severe falls. There was no end to the variety of postures which she assumed and motions which she executed. She did not sleep, and was emaciating.

"I now ordered tartar-emetic ointment to be rubbed upon the scalp, two or three times a day, until a copious eruption should appear. From the furious and restless state of the patient, the difficulty of using friction was very great. Some time, therefore, elapsed before a crop of pustules was produced; but upon their appearance the most decided improvement became evident. She now slept for several hours with only occasional starting, the involuntary motions were greatly abated, the countenance lively, the pulse full and soft, the skin, which formerly had been harsh and dry, was relaxed and moist, and the external senses were more alive to impressions. The pain of the occiput was gone, except what arose from the eruption, which was very great in this case, from her mother, disappointed by its not appearing so soon as was expected, having used the ointment too freely. Several small portions of the scalp sloughed. The ointment was the same (ʒj. to ℥j.) which the late Dr. Monteath and Mr. Mackenzie generally used as a counter-irritant in the treatment of the different ophthalmiæ, &c. in the Eye Infirmary. From this period she rapidly improved, all the functions returning gradually to their healthy state. The discharge from the scalp was maintained for some time by the occasional application of a little of the ointment, and when it began to decrease, the spine was rubbed with a liniment of turpentine and camphor, to hasten the cure, and prevent the danger of relapse. In ten days after the appearance of the eruption, she could walk through the house with a firm step, and handle even the smallest object: the pupils became more and more lively in their motions; the corneitis was completely dissipated and the vision improved. I now discontinued my attendance, but ordered the occasional use of the cold affusion, followed by dry friction with a coarse towel, and some tonic medicines, which she was to continue although all the symptoms were gone. I saw her about three weeks ago in the best health. There had not been the slightest appearance of any relapse."

We shall not follow Dr. Harrower through the long chain of remarks which he makes, and which are principally copied from Good's Study of Medicine. We shall only advert to his opinion, that the brain is the seat of chorea. "A very strong proof that the brain is the actual seat of chorea is, that when it arises from causes more particularly affecting that organ, it is more difficult of removal than when the brain is only secondarily affected, in consequence of some other organ labouring under disease." There can be no doubt that the disease often originates in the mind, the body becoming secondarily affected. In most cases, however, we find the alimentary canal greatly disordered, and to this we are obliged to direct our principal attention. In one of the three cases to which we have alluded, the slightest pressure on one particular part of the spine, or rather of the long muscles on one side of the spine, excited choreal movements in various parts of the body, and especially in one of the arms.

We have found a series of tartar-emetic plasters along the spine, together with purgatives, combined with the nitrate of silver, the most effectual plan of treatment. A grain of nitrate of silver, with five grains of the compound extract of colocynth, and two of the pilula hydrargyri, was given to one patient for several weeks, by which the alvine excretions were brought to a natural condition from the most vitiated state we have ever beheld. The power of walking increased as the secretions and excretions improved.

